# Cornerstone Cellular Pathways for Metabolic Disorders and Diabetes Mellitus: Non-Coding RNAs, Wnt Signaling, and AMPK

**DOI:** 10.3390/cells12222595

**Published:** 2023-11-09

**Authors:** Kenneth Maiese

**Affiliations:** Cellular and Molecular Signaling, New York, NY 10022, USA; wntin75@yahoo.com

**Keywords:** aging, AMP-activated protein kinase (AMPK), apoptosis, autophagy, erythropoietin, nicotinamide, non-coding RNA, oxidative stress, Wnt1 inducible signaling pathway protein 1 (WISP1), Wnt

## Abstract

Metabolic disorders and diabetes (DM) impact more than five hundred million individuals throughout the world and are insidious in onset, chronic in nature, and yield significant disability and death. Current therapies that address nutritional status, weight management, and pharmacological options may delay disability but cannot alter disease course or functional organ loss, such as dementia and degeneration of systemic bodily functions. Underlying these challenges are the onset of aging disorders associated with increased lifespan, telomere dysfunction, and oxidative stress generation that lead to multi-system dysfunction. These significant hurdles point to the urgent need to address underlying disease mechanisms with innovative applications. New treatment strategies involve non-coding RNA pathways with microRNAs (miRNAs) and circular ribonucleic acids (circRNAs), Wnt signaling, and Wnt1 inducible signaling pathway protein 1 (WISP1) that are dependent upon programmed cell death pathways, cellular metabolic pathways with AMP-activated protein kinase (AMPK) and nicotinamide, and growth factor applications. Non-coding RNAs, Wnt signaling, and AMPK are cornerstone mechanisms for overseeing complex metabolic pathways that offer innovative treatment avenues for metabolic disease and DM but will necessitate continued appreciation of the ability of each of these cellular mechanisms to independently and in unison influence clinical outcome.

## 1. The Impact of Metabolic Disorders in the Global Population

It is estimated that by the year 2045, almost eight hundred million individuals will suffer from metabolic disorders with the greatest majority of these individuals having diabetes mellitus (DM) [1]. These observations suggest that almost one in eight people will have DM, and this represents a fifty percent increase from the current prevalence of metabolic disorders. Presently, there are approximately five hundred thirty-seven million people with DM, and these numbers will increase to more than six hundred forty million individuals by the year 2030 [2,3,4,5,6]. Almost seventy-five percent of adults currently suffer from DM in predominately low- and middle-level income nations [1,7,8]. At least two million deaths a year from DM occur in relatively young people less than seventy years old [5,8,9,10,11,12] (Table 1).

DM is a chronic disorder that can impact all systems of the body. In particular, DM can lead to neuronal injury [13,14,15,16,17,18,19], dementia and memory loss [8,13,15,19,20,21,22,23,24,25,26,27,28,29,30], vascular disease [2,9,31,32,33,34,35,36,37,38,39], mitochondrial dysfunction [2,3,7,10,40,41,42,43,44,45,46], kidney failure [24,45,47,48], liver degeneration [41,45,49,50,51,52,53,54], and neurodegenerative disorders such as Alzheimer’s disease (AD), Parkinson’s disease (PD), and Huntington’s disease [18,22,25,27,28,40,55,56,57,58,59,60,61]. Although a chronic disorder, DM also can lead to acute disability and dysfunction with the onset of stroke [57,62,63], reductions in cerebral blood flow [8,28,36,44,63,64,65], increased sensitivity to infections such as with severe acute respiratory syndrome coronavirus (SARS-CoV-2) and coronavirus disease 2019 (COVID-19) [16,42,66,67,68,69,70,71,72,73], disease of the retina [37,39,74,75,76], stem cell impairment [2,77,78,79,80,81,82,83], dysregulation of the immune system [16,41,42,75,82,84,85,86,87,88,89,90], and cardiac disease [2,3,44,91,92,93,94,95].

In addition to the broad range of disorders that can result from metabolic disease, a significant financial burden to address metabolic disorders also is a recognized burden for both individuals and nations. The necessary finances to provide care for DM are increasing and presently estimated at USD seven hundred sixty billion [1]. An additional USD seventy billion is believed to be required for patients with significant disability. As a result, DM care consumes more than seventeen percent of the United States (US) gross domestic product [96]. At the individual level, every year USD twenty thousand is required to assist with glucose monitoring, infections, care coaching, and treatment for wounds [5,9,27,33,36,38,40,89,97,98,99,100,101]. Yet, these numbers do not fully grasp the overall financial requirements for metabolic disease and DM. Worldwide, four hundred million people currently have DM or are at significant risk of acquiring DM [1,59,102]. More than seven million individuals may suffer from DM but remain undiagnosed. In addition, over thirty-five percent of people in the US may be pre-diabetic with elevated fasting glucose and hemoglobin A1c (HbA_1c_) levels but are not currently under treatment [8,103].

## 2. Lifespan, Aging, Obesity, and Socioeconomic Status Can Impact Metabolic Disorders

Multiple factors can affect the development of metabolic disorders and DM, including increased lifespan and age of the population, lower levels of physical activity, increased weight with obesity, education level, and socioeconomic status (Table 1). Throughout the world, lifespan has been increasing to the extent that most individuals will reach at least eighty years of age [104,105,106,107,108,109,110]. Individuals in developing nations are expected to experience a rise in the number of those over the age of sixty-five to increase by ten percent, and in developed nations, individuals over the age of sixty-five have more than doubled over the prior fifty-year period [106,111]. A number of considerations have led to improved lifespan, including improved sanitation and environmental measures, early access to healthcare, broader public healthcare policies, and more effective nutrition programs [104,112,113,114]. However, with the increase in lifespan comes the effects of aging and degenerative processes. The onset of cell senescence and degeneration of tissues can occur with DM and metabolic dysfunction [2,3,4,41,79,82,115,116]. Destabilization of telomeres (TLs) through processes of shortening ultimately leads to cellular senescence [117,118,119,120]. TLs are formed from deoxyribonucleic acid (DNA), exist on chromosome ends, and control cell survival, DNA maintenance, and cell reproduction [2,3,118,120,121,122,123]. During cell replication, the telomerase protein lays down tandem repeat ribonucleic acid (RNA) templates to block base pair loss in TLs [117,118,122]. Yet, once TLs have less than five hundred base pairs, telomerase cannot maintain TL function, and the senescence of cells results [14,109,124,125,126,127,128,129,130,131,132,133,134]. With the onset of cellular senescence, repair of organs cannot take place, degeneration due to aging can begin, and the immune system also loses function to protect against environmental toxins [4,41,104,109,122,124,126,127,132,134,135,136,137,138,139]. In addition, TL shortening and the onset of cell senescence foster the production of reactive oxygen species (ROS) and oxidative stress that affects metabolic function and mitochondrial integrity [2,3,4,14,41,124,127,128,129,132,133,134,136,138,140,141,142,143,144,145].

**Figure 1:**
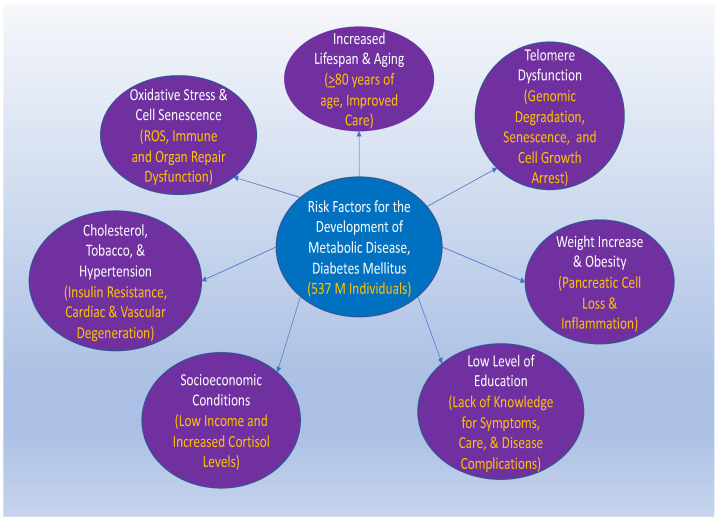
Multiple factors can influence the development of metabolic disease and diabetes mellitus. Factors that involve increased lifespan (≥80 years of age, improved care), aging, increased weight with obesity, lower education level, and socioeconomic status can have significant roles in the development of diabetes mellitus (DM) that affects 537 million (M) individuals. With aging-related disease, the destabilization of telomeres (with genomic degradation, senescence, and cell growth arrest) through processes of shortening ultimately leads to cellular senescence, oxidative stress (release of reactive oxygen species (ROS), and the degeneration of tissues and organs (with immune and organ repair dysfunction). In addition, other conditions that can be influenced by socioeconomic conditions (low income and increased cortisol levels) include elevations in serum cholesterol, high blood pressure, and tobacco use (insulin resistance and cardiac and vascular degeneration). A low level of education (a lack of knowledge of symptoms, care, and disease complications) and increased weight and obesity (pancreatic cell loss and inflammation) also impact DM.

Increased weight and obesity, level of education, and socioeconomic status also play a significant role in the development of DM. High body weight affects insulin sensitivity and glucose tolerance during DM [4,10,18,41,46,72,85,97,100,146,147,148,149,150,151,152,153,154,155,156,157,158,159,160]. Obesity can lead to impairments in stem cells, mitochondria, and the immune system, and also release ROS [2,10,14,46,72,85,146,155,161,162,163,164,165,166,167,168,169]. Obesity also can increase susceptibility to infection, such as with COVID-19 in patients with DM [170,171]. A low level of education, which can be affected by socioeconomic status, can lead to poor lifestyle habits and inadequate nutritional care, which leads to the development of DM. Individuals with higher education, such as more than a high school level, comprise about seven percent of people with DM, but those that have an education level below high school comprise about thirteen percent of people with DM [8,68,98,163,172]. Additional risks for the development of DM that may also be influenced by environmental and socioeconomic factors, including elevations in serum cholesterol, high blood pressure, raised cortisol levels, and tobacco use [2,21,45,66,152,166,173] (Figure 1). 

## 3. The Need for Clinical Innovation for the Treatment of Metabolic Disease and Diabetes Mellitus

**Figure 2:**
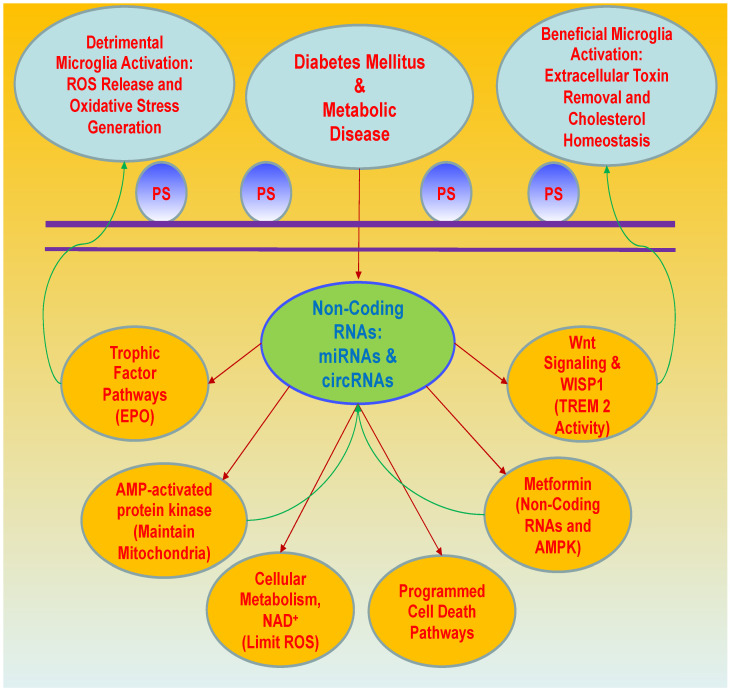
New treatment strategies for metabolic disease and diabetes mellitus with non-coding RNAs. Non-coding RNAs play a central role in the oversight of complex metabolic pathways that offer innovative treatment avenues for metabolic disease and diabetes mellitus (DM). Innovative considerations involve non-coding RNA pathways with microRNAs (miRNAs) and circular ribonucleic acids (circRNAs), Wnt signaling, and Wnt1 inducible signaling pathway protein 1 (WISP1) that are dependent upon programmed cell death pathways, such as apoptosis and the externalization of membrane phosphatidylserine (PS) residues on cell membranes, cellular metabolic pathways with AMP-activated protein kinase (AMPK) and nicotinamide adenine dinucleotide (NAD+) pathways with nicotinamide, and growth factor applications. These pathways intersect with one another for new therapeutic strategies, such as controlling microglial activation and limiting reactive oxygen species (ROS) generation. Microglia can be detrimental to the release of reactive oxygen species (ROS) to generate oxidative stress but also can be beneficial for the clearance of toxins (amyloid) in the brain and the reduction of inflammation. Importantly, microglial pathways are overseen by Wnt signaling and erythropoietin (EPO). Triggering receptor expressed on myeloid cells 2 (TREM2) is vital to foster microglial survival to prevent inflammation. In addition, metformin, as well as trophic factors with EPO, as examples of new therapeutic strategies, can reduce metabolic dysfunction and assist with the treatment of dementia, cardiovascular disease, multiple sclerosis, and peripheral neuropathy through the oversight of microglia, AMPK (maintains mitochondrial function), and non-coding RNA pathways.

Metabolic disorders and DM present multiple challenges for both patients and clinicians. Disorders, such as DM, are chronic in nature, insidious in onset, progressive without resolution, and lead to significant disability and death for a large proportion of the global population. DM affects all systems of the body resulting in numerous disorders that can include cardiovascular disease, neurodegenerative disorders, renal disease, hepatic disease, and musculoskeletal disease [2,3,8,15,25,40,41,44,47,48,50,51,52,57,59,66,91,92,93,149,163,172,174,175]. Although the observed increase in lifespan and improved access to medical care may be a welcome assistance to patients and clinicians that can address some risk factors involving nutrition, diet and weight management, hypertension, cholesterol levels, and tobacco use, current treatments that also involve pharmaceutical care cannot always control frequent periods of hyperglycemia or hypoglycemia [2,3,4,5,9,10,27,32,50,67,176] (Figure 1). These fluctuations in glucose homeostasis may result in decreased cell survival in multiple organs and lead to organ degeneration [14,101,177,178]. Even if disorders that involve loss of brain mass are addressed, with present therapies, cognitive impairment can progress without abatement [59,172,179]. Furthermore, the global increase in lifespan has led to aging-related processes with TL dysfunction, cellular oxidative stress, and cellular senescence that can increase the risk for organ failure in the setting of metabolic disorders. These significant challenges point to the need for new innovative insights into the clinical care of patients with metabolic disorders and DM to address underlying disease mechanisms. Novel considerations for the development of new strategies for metabolic disorders and DM involve non-coding RNA pathways with microRNAs (miRNAs) and circular ribonucleic acids (circRNAs), Wnt signaling, and Wnt1 inducible signaling pathway protein 1 (WISP1) that are intimately tied to aging-related disease, oxidative stress, programmed cell death pathways, cellular metabolic avenues with AMP-activated protein kinase (AMPK) and nicotinamide, and trophic factor considerations (Figure 2).

## 4. Cellular Mechanisms of Oxidative Stress, Energy Metabolism, and Programmed Cell Death with Metabolic Disorders 

In the presence of metabolic disease and DM, aging processes with the shortening of TLs and the presence of risk factors with obesity can foster the generation of ROS and oxidative stress (Table 1). Oxidative stress during DM can influence cell survival, cellular organelle integrity, and pathways that affect programmed cell death [5,19,25,33,42,49,63,71,85,86,168,180,181,182,183,184,185]. Oxidative stress occurs during the generation of ROS that can be formed by entities that include superoxide free radicals, peroxynitrite, singlet oxygen, nitric oxide, and hydrogen peroxide [80,164,185,186,187,188,189,190,191]. With the onset of oxidative stress and the release of ROS, injury can occur to neurons [15,19,76,191,192,193,194,195,196,197,198,199,200,201], vascular cells [33,36,38,190,202,203,204,205,206,207], stem cells [80,199,208,209,210], hepatic cells [51,53,211,212,213,214,215,216], renal cells [15,43,217,218,219,220], and musculoskeletal cells [36,152,221,222,223,224]. Intrinsic systems in the body that comprise catalase, glutathione peroxidase, vitamins B, K, E, D, and C, and superoxide dismutase can assist in limiting oxidative stress but can be overwhelmed during disease states, such as DM [5,7,42,43,44,114,134,160,225,226,227,228,229,230,231,232,233]. 

Of the potential systems that can limit oxidative stress, especially during metabolic disorders, is the vitamin nicotinamide, which is an interesting consideration (Figure 2). Nicotinamide is the amide form of vitamin B_3_ (niacin) and is obtained from plant or animal sources as well as dietary supplements [7,45,81,234,235,236,237,238,239,240,241,242,243]. Nicotinamide can be formed from a change in nicotinic acid (the water-soluble form of vitamin B_3_) in hepatic cells or by coenzyme ß-nicotinamide adenine dinucleotide (NAD^+^) hydrolysis [42]. Once present, nicotinamide is required for nicotinamide adenine dinucleotide phosphate (NADP^+^) generation and is a precursor for NAD^+^ [7,81]. Loss of adequate cellular levels of nicotinamide can lead to oral ulcerations, pigmentation of the skin, pellagra, appetite loss, inflammation, infection, and fatigue [20,238,241,242].

As a protective entity that can be considered an anti-oxidant during metabolic disease [5,164,199,244,245,246,247,248,249,250,251,252], nicotinamide can increase survival during oxidative stress for neuronal cells [194,234,253,254] and endothelial cells [7,203,243,255,256,257] and also promote energy maintenance of mitochondrial function [2,7,140,141,242,258,259,260,261,262]. Nicotinamide can block apoptotic cytochrome c release through the control of mitochondrial pore generation [239,254,255]. In addition, nicotinamide can prevent the depolarization of mitochondrial membranes through phosphorylation of the BCL2-associated agonist of cell death (BAD) [239,254,255], maintain stability for mitochondrial membrane potential [194,242,255,261,263,264], and inhibit mitochondrial permeability transition pore complex assembly [265]. During metabolic disorders, nicotinamide can modulate insulin resistance and glucose homeostasis [49,81,240,266]. Nicotinamide also can alleviate the activation of inflammatory pathways [20,140,239,241,267,268,269] and may limit muscle degeneration [137,262,270,271]. It should be noted that protection with nicotinamide whether in cell models or higher organisms requires a specific concentration range. In fact, higher concentrations of nicotinamide have been demonstrated to be harmful [7,264,272] or may affect pathogen virulence [273].

The pathways of programmed cell death during DM are closely linked to cellular energy pathways and oxidative stress (Figure 2). Apoptosis yields cell death during metabolic disease through a series of stages [106,159,274,275,276]. The initial stage involves the externalization of membrane phosphatidylserine (PS) residues on cell surfaces that can attract inflammatory cells, such as microglia, to dispose of injured cells during the initial phase of apoptosis [106,131,277,278,279,280,281]. This initial stage of apoptosis is potentially reversible by preventing the externalization of membrane PS residues [277,282,283,284,285] to block inflammatory cells from detecting and removing injured cells that may remain functional [76,286,287,288,289]. If the second phase of apoptotic cell death is reached, it is usually not reversible and involves the degradation of nuclear deoxyribonucleic acid (DNA) [136,137,167,169,290,291,292,293,294]. The second stage of apoptosis consists of mitochondrial membrane depolarization, cytochrome c release, and caspase activation [136,193,252,290,295,296,297,298,299,300]. Apoptosis during DM can lead to atherosclerotic plaque generation [296,301,302], foster processes associated with infection, such as COVID-19 [59,71,303,304], promote joint degenerative diseases [152,293,305,306,307], and enhance stem cell demise and inflammatory pathway activation [26,103,129,132,291,308,309,310,311,312,313,314]. Apoptosis during metabolic disorders also can be involved in adipose tissue inflammation during loss of metabolic homeostasis [315], may lead to cognitive loss in combination with autophagic pathways [77], promote microglial activation to the detriment of cells [26], impair pancreatic β-cell function [61,316], promote demyelination of nerve fibers [317], lead to ischemic cell injury [63], result in retinal cell loss [75,76,292,318,319,320,321], foster renal cell injury [43,220,322], and lead to vascular cell degeneration [53,101,215,277,323]. In particular, microglia are important for removing damaged cells during membrane PS externalization and apoptosis [106,131,145,277,278,298,308,324,325,326,327]. Yet, microglia can lead to the generation of oxidative stress through the production of ROS [8,165,167,246,250,328,329,330,331], which can require modulation by non-coding RNAs [251,332,333,334,335,336], Wnt signaling [27,28,106,115,276,337,338,339], and trophic factor pathways with erythropoietin (EPO) [27,340,341,342,343,344,345,346]. In other scenarios, microglial cells can be helpful for protection during amyotrophic lateral sclerosis [347], remove brain amyloid [348,349], and preserve cholesterol homeostasis with autophagy [327]. As a pathway that can lead to increased survival for microglia, triggering receptors expressed on myeloid cells 2 (TREM2) can block inflammation during AD, which also may require Wnt signaling [350,351]. Interestingly, TREM2, similar to the neurofilament light chain, may function as a biomarker to signal early disease progression during AD and PD [350,352,353].

Autophagy is another programmed cell death pathway that is involved in the sequestration of cytoplasmic proteins and organelles for recycling and tissue remodeling [59,246,274,275,354,355,356,357,358]. Most descriptions of autophagy involve macroautophagy, rather than microautophagy or chaperone-mediated autophagy [59,359], which can form autophagosomes for combining into lysosomes. These cellular subunits will then be degraded and used to create new cellular components [111,132,360,361,362]. In several circumstances, autophagy activation can be protective during metabolic disorders and DM [7,16,28,40,43,53,75,81,88,93]. Exercise is an important consideration in the activation of autophagy since exercise programs can help reduce metabolic disease and may improve cognition [66,81,152,166,180,184,262,363,364,365,366,367]. In animal models, exercise can promote autophagy, maintain glucose homeostasis [368], raise insulin sensitivity [369], and promote microglial function during glucose cyclic changes [26]. Exercise may rely upon some portion of autophagy activation to generate mitophagic flux in the liver to maintain mitochondrial function during metabolic disease [50]. In addition, memory may be improved with low-calorie diets that promote autophagy [370]. Autophagy activation may be required for the function of circular RNAs during oxidative stress, inflammation, and insulin secretion [316] to prevent cerebral ischemia under conditions of DM [63] and limit retinopathy during DM [37]. The processing of circulating oxidized fatty acids during DM may require autophagy activation [18,165], and mitochondrial homeostasis can be dependent upon the activation of autophagy pathways [46]. Maintenance of autophagic flux during DM also may be one factor in preserving cognition [77,371], maintaining muscle integrity [354], fostering the function of pancreatic β-cells [372], decreasing insulin resistance in models of autophagy *Atg7* gene deletion and obesity [373], blocking nephropathy during DM with maintenance of autophagy Atg7, Atg5, and microtubule-associated protein 1A/1B-light chain 3 (LC3) proteins [374], and controlling the development of pancreatic β-cells [375]. Yet, a balance in autophagy activation is necessary since autophagy also can lead to detrimental effects. The activation of autophagy can lead to epididymal tissue injury during periods of hyperglycemia [376] and testicular demise with spermatogenic cell apoptosis [377]. During glucose fluctuations, microglial activity can be increased through inflammatory pathways that result in apoptotic cell loss mediated through autophagy [26], and memory loss may ensue through pathways of autophagy [22]. During the presence of advanced glycation end products (AGEs) and high glucose levels with autophagy, atherosclerosis [378], cardiac disease [379], and endoplasmic reticulum stress [380] can be present. Therapy designed to improve glucose regulation with the activation of autophagy may reduce heart and liver mass [177], reduce cerebral interneuron progenitor cell survival [381], foster death of neurons [382,383,384], enhance memory loss [132,362,385,386,387,388], and potentially injure mitochondria [46,50,178,197,220,235,343,366,389,390,391,392]. Cellular protection with growth factors, such as EPO, requires a reduction in autophagy activation in combination with the mechanistic target of rapamycin (mTOR), protein kinase B (Akt), the proline rich Akt substrate 40 kDa (PRAS40), and mammalian forkhead transcription factors [290,306,310,340,341,343,344,345,362,393,394,395,396,397,398,399].

Additional programmed cell death pathways, such as ferroptosis and pyroptosis, also can be important during metabolic disorders. Ferroptosis involves iron storage pathways that block glutathione homeostasis [8,232,233,249,400,401,402]. The loss of oxidative defenses that require glutathione during ferroptosis leads to memory loss [8,122,232], neuronal and glial cell dysfunction [59,172,400], cardiac impairment [2,403], osteoarthritis [233], and disorders in the tissue of the breast [169]. Pyroptosis, which can work in conjunction with necroptosis and apoptosis [41,59,269,304,404,405], leads to inflammatory cell activation, inflammasome generation, and caspase 4, caspase 5, and caspase 1 activation [8,221,311,404,406,407,408,409]. As a result of excessive cytokine release during pyroptosis [304,405], immune system activity is affected [409] and loss in cognitive function can occur [13,17,22,55,132,163,172,410,411]. 

## 5. Non-Coding RNAs, MicroRNAs, and Circular RNAs in Metabolic Disorders 

Small non-coding RNAs, termed miRNAs and circRNAs, play a critical role during metabolic disorders and DM [27,74,100,335,362,412,413,414] (Table 1). These small non-coding RNAs also are involved in programmed cell death pathways, such as apoptosis [21,128,305,307,313,415,416,417,418,419], autophagy [316,334,420,421,422,423,424,425], ferroptosis [2,426], and pyroptosis [172,427] (Figure 2). MiRNAs consist of 19–25 nucleotides that oversee the expression of genes by silencing or blocking messenger RNAs (mRNAs) that are targeted to translate specific genes into proteins [125,333,334,335,336,414,428,429,430,431,432,433,434,435,436,437]. In models of diabetic nephropathy, miRNA activation can block ferroptotic cell injury [426]. MiRNAs can also act to reduce oxidative stress and inflammation to protect insulin secretion in pancreatic β-cells [316]. The overexpression of a specific miRNA, miR-18a-3p, can alleviate cardiomyopathy during DM and prevent pyroptosis activation [427]. However, the activity of miRNAs during DM does not always lead to the protection of cells, and for specific miRNAs, inhibition may be the appropriate course for cellular protection. For example, miR-34a up-regulation can lead to endothelial dysfunction during oxidative stress and DM vascular disease [412,438]. This can be modulated by metformin to control miR-34a and mediate vascular protection [412]. In addition, EPO administration is necessary to prevent cell injury with miR-21 and other miRNAs [439].

CircRNAs consist of non-coding RNAs of approximately 100 nucleotides [100,142,152,313,316,329,333,334,335,336,414,419,432,440,441,442,443,444,445]. First identified as having a circular structure, circRNAs use covalent bonds to maintain a circular nature, contain both *cis* and *trans* regulation, oversee the expression of genes through the sponging of miRNAs [335,446,447,448], and can have value as biomarkers [329,335,429,432,436,449]. In vascular and metabolic disease, atherosclerosis may be prevented through circular antisense non-coding RNA in the INK4 locus (circANRIL) in vascular smooth muscle cells and macrophages by blocking exonuclease-mediated pre-ribosomal RNA generation and cell proliferation [450]. During periods of glucolipotoxicity in DM, circPIP5K1A can act as a sponge to reduce protective miRNA miR-552-3p and allow autophagy activation of pancreatic β-cells, suggesting that the down-regulation of circRNA circPIP5K1A can be a target for disease treatment [316]. As another example of potential detrimental outcomes with circRNAs, circRNA expression can reduce beneficial miRNA expression and yield excessive amyloid production in the brain [431] by down-regulating protective pathways of the silent mating type information regulation 2 homolog 1 (*Saccharomyces cerevisiae*) (SIRT1) [27,131,231,451,452,453,454,455]. Yet, applications with circRNAs also can have a beneficial outcome. Treatment with the circRNA CiRS-7 as a sponge for the miRNA miR-7 can promote insulin secretion and prevent the onset of DM [456]. Targeting circRNA also may be vital for the treatment of diabetic retinopathy [74], functioning as biomarkers for cardiovascular DM disease [100] and overseeing mTOR pathways with PRAS40 [53,335].

## 6. Wnt Signaling and WISP1 Oversight in Diabetes Mellitus and Metabolic Disorders 

Wnt signaling and WISP1 are vital pathways during metabolic disorders and DM for the oversight of oxidative stress, programmed cell death, and non-coding RNA function (Figure 2). Wnt proteins, which are cysteine-rich glycosylated proteins, are part of the *wingless* pathway that can modulate cell development and survival during aging, cardiovascular disorders, tumorigenesis, organogenesis, neurodegeneration, vascular disease, inflammation, and DM [25,28,48,76,92,115,131,150,184,276,296,297,305,307,338,339,457,458,459,460,461,462,463,464,465,466,467]. Wnt proteins that can involve Wnt1 oversee programmed cell death [105,219,278,299,339,362,387,467,468,469,470,471,472], pancreatic β-cell development and growth [473], skeletal function [152,305,307], trophic factor protection [323,439,474,475,476,477], and memory and executive function [76,362,478,479,480,481]. Wnt proteins can oversee vascular integrity and vascular calcification through pathways that are dependent upon SIRT1 and miRNAs, such as miR-126 [38,47]. Wnt signaling also can influence inflammation, angiogenesis, and leukostasis in retinal disease during DM [482,483]. During models of experimental DM, trophic factors that include EPO rely upon Wnt signaling for cellular protection [323,484]. Loss of Wnt signaling may foster the onset of DM [25], lead to cardiac injury during DM [92], vascular dysfunction [38], and metabolic neurodegeneration [14,28,48], and may be associated with central abdominal fat mass and adipose tissue dysfunction [76,98,149,150].

WISP1 is a downstream component of wingless signaling with Wnt proteins and a member of the CCN family of secreted extracellular matrix-associated proteins, six in number, that are termed by the first three members of the family that include cysteine-rich protein 61, connective tissue growth factor, and nephroblastoma overexpressed genes [14,27,299,485,486]. Similar to the Wnt1 signaling pathway, WISP1 can control the stability and progression of atherosclerotic vascular plaques [296], decrease through Akt pathways lipopolysaccharide-induced injury of cells [297], alter blood–brain barrier disease [485], protect neuronal survival [487,488], and limit oxidative stress [106,489,490]. During metabolic disease and DM, WISP1 is a marker of adipose tissue inflammation [491]. It is involved in pancreatic regeneration during glucose homeostasis [492] and can foster pancreatic β-cell development [493]. During periods of loss of glucose homeostasis, WISP1 may be protective since it is elevated during gestational DM [150,494] and has higher serum levels and insulin resistance in obese children and young adolescents [150,495,496] (Table 1). 

WISP1 modulates metabolic cellular pathways through AMPK and also has feedback mechanisms with itself and miRNAs. AMPK can assist with energy metabolism and lead to the production of adenosine triphosphate (ATP), which may influence sensory nerve function. For example, pain during DM with peripheral neuropathies can be relieved by AMPK activation in experimental models [497]. In addition, nicotinamide relies upon AMPK to preserve mitochondria function [261], and Wnt family members employ AMPK to limit neuronal injury [498]. AMPK can maintain electrical activity of the cortex for behavior control [499], AMPK oversees endothelial tight junctions [500], and AMPK can promote mitochondrial integrity during ferroptotic cell death [169]. In the absence of AMPK activity, cell senescence, cell death, and mitochondrial injury can ensue [3,137]. WISP1 controls the phosphorylation of AMPK by differentially limiting phosphorylation of tuberous sclerosis 2 (TSC2) at serine^1387^, a target of AMPK, and promoting phosphorylation of TSC2 at threonine^1462^, a target of Akt [103,300,310,345,396,490,501] that has been shown to mediate protection of pancreatic cells [502] and neuroprotection [60,455,503] through glucagon-like peptide-1 (GLP-1). This ability of WISP1 to target and control AMPK may improve cell survival and metabolic homeostasis [168] since AMPK at times can reduce oxidative stress, limit insulin resistance [369], and lower lipid accumulation [504]. Yet, AMPK has another side requiring close regulation since under other circumstances, AMPK may lead to cell demise with autophagy [8,505,506]. In addition, non-coding RNAs can indirectly control WISP1, signaling the modulation of AMPK. AMPK is independently linked to miRNAs and can control miRNA expression, such as miR-185, to offer cellular protection through the up-regulation of miR-185 [425]. Furthermore, miRNAs, such as miR-185, can suppress AMPK and autophagy activity to lead to increased cell survival and block apoptosis [425]. Under conditions with growth factors, such as EPO, AMPK activity must be regulated to limit oxidative stress [490] and inflammation [306,507] since the elevated activity of EPO and AMK can result in cell injury [508]. EPO can modulate AMPK activity [27,509,510,511,512]. Interestingly, WISP1 can control its own expression through autophagy and apoptotic pathways [513]. In addition, WISP1 can be regulated through potential feedback mechanisms involving miRNAs, such as miR-515-5p and miR-128-3p, to have WISP1 expression reduced [305,307] for improved glucose homeostasis. In other considerations with WISP1, the down-regulation of WISP1, either through the direct control of non-coding RNAs or through non-coding RNAs using AMPK, may be a potential therapeutic target to limit tumor growth [514], an important consideration for the trophic pathways of Wnt signaling and WISP1 that can promote tumorigenesis [76,130,337,457,461,462,470,515,516].

## 7. Conclusions and Future Perspectives

Metabolic disorders and DM are chronic diseases that affect a significant number of individuals in the global population. It is expected by the year 2030, more than 640 million individuals will be affected by DM, and the greatest proportion of these individuals reside in low- and middle-income nations. Financial considerations to care for individuals with metabolic disease are equally staggering and can exceed USD seven hundred billion with at least USD twenty thousand required annually for every patient to provide minimum care including glucose monitoring, nutritional coaching, and treatment of infections and wounds. Yet, these challenges may not provide the complete picture since more than four hundred million people may remain currently undiagnosed with either pre-diabetes of DM and have elevated fasting glucose and HbA_1c_ levels.

DM affects all systems of the body and can lead to renal failure, liver disease, neurodegeneration with cognitive loss, and cardiovascular disease. Furthermore, metabolic disorders and DM are chronic and progressive in nature that lead to severe disability and death. Underlying these conditions are the effects of aging, increased weight gain, and additional risk factors that can be tied to socioeconomic status. With the observed increase in global lifespan, the consequences of aging in the presence of metabolic disease and DM can involve TL dysfunction, the onset of cellular senescence, and organ and tissue degeneration. Accompanying these processes are the increased risk of obesity that leads to immune system dysfunction, infection susceptibility, such as with SARS-CoV-2 and COVID-19, loss of mitochondrial integrity, and the generation of oxidative stress. Lower socioeconomic status compounds these complications for the development of DM, which include low education status and inadequate nutritional care. Current therapies for DM attempt to address these risk factors with access to proper nutritional education and weight management that are accompanied by pharmaceutical agents to manage insulin release and resistance as well as overall glucose homeostasis. Yet, these strategies do not halt overall disease progression and can lead to disability with periods of hypoglycemia or hyperglycemia. These therapies also may lead to decreased cell survival in multiple organs, promote the degeneration of organs, and may have no effect on the progression of disorders involving cognitive loss in the central nervous system and nerve degeneration in the peripheral nervous system. Such considerations demand innovative clinical strategies to address the underlying mechanisms of metabolic disorders and DM that involve non-coding RNA pathways with miRNAs and circRNAs, Wnt signaling, and WISP1. These pathways are intimately tied to the generation of aging pathways, ROS, and oxidative stress and can function through programmed cell death mechanisms, metabolic pathways involving AMPK and nicotinamide, and trophic factor applications.

Oxidative stress is a critical pathway in the pathology of metabolic disorders and DM. The release of ROS can lead to the death of multiple cell types, which can affect neurons, vascular cells, stem cells, and musculoskeletal cells. The presence of intrinsic and extrinsic anti-oxidant systems can offer a vital aide to potentially prevent both the onset and progression of DM. Nicotinamide can be an important component of these anti-oxidant systems, especially as a precursor for NAD^+^, offering the ability to maintain cellular metabolic homeostasis. Nicotinamide can be effective against oxidative stress and offer cellular protection at a number of levels in the apoptotic death cascade, which include maintenance of mitochondrial membrane potential, reduced activity of inflammatory pathways, limited muscle degeneration, and assistance with insulin resistance. Yet, concentrations of cellular nicotinamide should always be considered since elevated levels of nicotinamide can decrease SIRT1 activity [517] and may conceivably reduce protection for cells during oxidative stress through the loss of SIRT1. In addition, elevated concentrations of nicotinamide can lead to decreased cell survival through other means.

The pathways of programmed cell death are also important targets for the treatment strategies of metabolic disorders and DM. Therapies that can address early phases of apoptosis during membrane PS residue externalization can be reversible and could protect impaired pancreatic β-cells, prevent retinal cell disease and neurodegeneration, control activation of inflammatory cells, such as microglia, and protect against cardiovascular disease. The use of proteomics also may assist with the investigation of these pathways with apoptosis, which examines the function and cellular activities of proteins at the cellular level. For example, the understanding of intracellular and extracellular apoptotic protein bodies can provide insight into metabolic, neurodegenerative, aging, and cancer pathways [50,304,518,519,520,521]. Autophagy is closely tied to apoptotic pathways and can limit retinopathy during DM, foster the processing of circulating oxidized fatty acids, maintain mitochondrial integrity, especially during exercise, oversee the development and function of pancreatic β-cells, and preserve memory function. However, the modulation of autophagy pathways requires a careful balance since clinical strategies that incorporate autophagy for glucose homeostasis can ultimately, if left unchecked, lead to loss of organ mass, atherosclerosis development, decreased interneuron progenitor cell survival, neuronal cell death, mitochondrial dysfunction, and cognitive loss. In addition, serum glucose fluctuations can promote inflammatory pathways mediated by autophagy that result in apoptotic cell death and also activate mechanisms of pyroptosis and ferroptosis. Growth factors, such as EPO, also require the down-regulation of autophagy pathways for neuronal and vascular protection in DM.

Interestingly, programmed cell death pathways work in conjunction with non-coding RNAs, Wnt signaling, and WISP1. The activation of autophagy is necessary for the function of circRNAs in the modulation of oxidative stress, inflammatory activation, and the secretion of insulin. In fact, non-coding RNAs are involved with almost all types of programmed cell death pathways, including apoptosis, autophagy, ferroptosis, and pyroptosis. Both microRNAs and circRNAs can limit oxidative stress and inflammation, oversee insulin secretion, act as biomarkers, and prevent atherosclerotic disease. Depending on the specific nature of the non-coding RNA and the relationship between microRNAs and circRNAs functioning as sponges, either enhanced cellular survival can be fostered or detrimental outcomes may result, such as the excessive deposition of amyloid in the brain. Studies are employing regularly interspaced palindromic repeats (CRISPR) and CRISPR-associated protein 9 (Cas9) technology to further elucidate the role of non-coding RNAs [444], inflammation [522], and Wnt signaling [485,522] in experimental models. For these reasons, the role of Wnt signaling and WISP1 becomes critical since non-coding RNAs can modulate the function of these pathways. Wnt signaling and WISP1 offer a number of protective outcomes during metabolic disease and DM, including controlling programmed cell death pathways, overseeing glucose homeostasis through AMPK-mediated pathways, limiting oxidative stress generation, promoting vascular cell integrity and reducing atherosclerosis, fostering pancreatic regeneration and pancreatic β-cell development, and assisting with insulin resistance. However, Wnt signaling and WISP1, as strong trophic-based pathways, can promote tumorigenesis. As a result, oversight of Wnt signaling and WISP1 through non-coding RNAs becomes a critical element as an essential feedback mechanism in these pathways when considering clinical applications for the treatment of metabolic disease and DM. In addition, non-coding RNAs can indirectly regulate WISP1 signaling through AMPK to either control the activity of AMPK or promote the ability of AMPK to control miRNA expression and activity. 

It is also important to note the direction of current and future strategies of treatment for these pathways for metabolic disorders, including pharmaceuticals, cell-based therapies, and biological factors. In this regard, AMPK pathways are central to present agents to treat DM; they include metformin and biguanides to reduce the effects of neurodegenerative and vascular disease. These include therapies for dementia, cardiovascular disease, multiple sclerosis, and peripheral neuropathy [2,42,59,71,172,240,523,524]. Metformin can reduce metabolic dysfunction and lipid peroxidation in the brain and spinal cord by reducing caspase activity to promote the survival of cells [525]. Metformin also controls glucolipid metabolism [159], reduces aging-related disorders [4,5], limits inflammation [111,252,359,526], and blocks TOR activity to increase autophagy induction, and it may provide increased cell survival at times independent of AMPK [527]. Metformin is being considered for other disease applications, such as limiting disability in patients with obesity or individuals with DM during coronavirus disease 2019 (COVID-19) [42,70,71,170,171,172], as well as increasing the recovery of myelin in experimental models of multiple sclerosis [523]. Of note, metformin is vital for the control of non-coding RNAs as well. It has been shown that miR-34a up-regulation can lead to endothelial dysfunction during oxidative stress and DM. Yet, this disease process can be modulated by metformin to oversee miR-34a and foster vascular protection. Microglia also represent an emerging target for therapy in metabolic disease. Microglia are vital for removing injured cells, especially those tagged by membrane PS externalization, but careful modulation of these inflammatory cells is important since on one hand, they can lead to the production of ROS and oxidative stress that can result in the loss of metabolic homeostasis. Yet, on the other hand, microglia can assist with the removal of toxins, such as amyloid, and preserve cholesterol homeostasis with autophagy. Through pathways that are dependent upon TREM2, new therapies using microglia are being considered to improve cognition, reduce memory loss, block inflammation, and be important tools for identifying metabolic disease progression. The therapeutic pathways that oversee microglia function are intimately tied to non-coding RNAs that oversee inflammatory pathways [251,332,333,334,335,336], Wnt signaling, and growth factors, such as EPO. Growth factors, such as EPO, are also being seen as necessary to control AMPK and non-coding RNA pathways. Independently, pathways, such as Wnt signaling and WISP1, are now being considered biomarkers for the risk of gestational DM and tissue inflammation in DM. In summary, non-coding RNAs with miRNAs and circRNAs have a vital oversight of each of these complex metabolic pathways, which involve aging processes, oxidative stress, programmed cell death pathways, Wnt signaling, WISP1, AMPK, and cellular metabolism pathways and can involve nicotinamide and trophic factors. As a result, non-coding RNAs, Wnt signaling, and AMPK offer exciting considerations for the future development of innovative strategies for metabolic disorders, but a further appreciation of the multifarious relationship among these cellular pathways is necessary for the effective execution of clinical care.

## Figures and Tables

**Table 1:** Highlights of implementing strategies for non-coding RNAs with microRNAs and circular RNAs in metabolic disorders and diabetes mellitus.

Metabolic disorders and diabetes mellitus (DM) are insidious in onset, progressive in nature, chronic in duration, and are expected to impact one in eight individuals, and over USD eight billion are necessary on an annual basis to meet clinical needs.
Multiple factors can influence the development of metabolic disorders and DM, including increased weight with obesity, lower education levels, socioeconomic status with limited health resources, and increased lifespan with age-related disease, telomere dysfunction, cellular senescence, generation of reactive oxygen species (ROS), and tissue and organ degeneration.
Given that current therapies for metabolic disease and DM are not curative for these disorders, innovative treatment avenues are required that involve non-coding RNA pathways with microRNAs (miRNAs) and circular ribonucleic acids (circRNAs), Wnt signaling, and Wnt1 inducible signaling pathway protein 1 (WISP1) that are linked to programmed cell death pathways, oxidative stress, cellular metabolic pathways with AMP-activated protein kinase (AMPK) and nicotinamide, and growth factor applications.
Non-coding RNA pathways with miRNAs and circRNAs play a central role in the oversight of programmed cell death pathways, Wnt signaling, WISP1, and AMPK to offer mechanisms for pancreatic β-cell protection, reduction in inflammatory pathways, maintenance of mitochondrial integrity, promotion of insulin secretion, reduction in insulin resistance, and enhancement of cellular survival.
Yet, detailed insight into non-coding RNA and related pathways is critical for the development of future clinical applications since these pathways are intimately linked to one another, have complex autofeedback systems, and can sometimes lead to detrimental outcomes such as the destruction of pancreatic β-cells, loss of glucose homeostasis, and distant systemic organ effects, such as excessive amyloid deposition in the brain.
